# Novel insights into the bone microenvironment in myeloma bone disease

**DOI:** 10.1016/j.jbo.2026.100815

**Published:** 2026-11-03

**Authors:** Franziska Jundt, Wen-Hui Cheng, Poonam Kumari, Heike Weidner, Luise Fischer, Maximilian Merz, Andreas Beilhack, Martin Kuric, Regina Ebert

**Affiliations:** a Department of Internal Medicine II, University Hospital Würzburg, Oberdürrbacher Straße 6, 97080 Würzburg, Germany; b Division of Endocrinology, Diabetes, and Bone Diseases, Department of Medicine III & Center for Healthy Aging, Medical Faculty and University Hospital Carl Gustav Carus, Dresden University of Technology, Germany; c Department of Hematology, Cell Therapy, Hemostaseology, and Infectious Diseases, University Hospital, Leipzig, Germany; d Myeloma and Cellular Therapy Services, Memorial Sloan Kettering Cancer Center, New York, NY, USA; e Department of Internal Medicine II, Center for Experimental Molecular Medicine, University Hospital Würzburg, Zinklesweg 10, 97080 Würzburg, Germany; f Department of Musculoskeletal Tissue Regeneration, University of Würzburg, Friedrich-Bergius-Ring 15, 97076 Würzburg, Germany

**Keywords:** Multiple myeloma, Myeloma bone disease, Bone microenvironment, Osteocytes, Osteolytic lesions, Extracellular matrix, Matrisome, Mechanobiology, Mechanical loading, Bone remineralization, Lacuno-canalicular network, Bone repair, CAR-T-cell therapy

## Abstract

Myeloma bone disease develops in patients with a monoclonal expansion of malignant plasma cells in the bone marrow that induce osteoclast activity and suppress osteoblast function. Consequently, osteolytic bone lesions occur, causing fractures, pain, and limiting quality of life. Recent experimental, imaging, biomaterial, and translational studies indicate that multiple myeloma remodels the bone microenvironment at several interconnected levels: the extracellular matrix and matrisome, adhesion-dependent microenvironmental interactions, the immune contexture of the bone microenvironment, osteocyte lacunar architecture, bone material quality, mechanosensing, and the capacity for remineralization. Emerging evidence further suggests that endothelial adhesion molecules such as junctional adhesion molecule-A (JAM-A) and spatially organized immune-suppressive niches contribute to angiogenesis, tumor dissemination, immune escape, and persistence within osteolytic bone lesions. The emerging concept is that the myeloma bone niche is a structurally, mechanically, and molecularly altered ecosystem that both supports tumor growth and limits skeletal repair. In parallel, preclinical concepts suggest that the myeloma bone niche can be modulated by biophysical stimuli to induce skeletal repair in myeloma patients. Additionally, increasing evidence indicates that anti-myeloma therapies, including proteasome inhibitors, immunomodulatory agents, monoclonal antibodies, and B-cell maturation antigen (BCMA)-directed chimeric antigen receptor (CAR) T-cell approaches, not only control tumor burden but also modulate bone remodeling through direct effects on osteoclasts and osteoblasts as well as indirect reprogramming of osteoimmune interactions within the bone marrow microenvironment. While some of these therapies promote bone formation or reduce resorption, their overall impact on skeletal repair remains heterogeneous and incompletely understood.

## Highlights

• Myeloma bone disease reshapes the bone microenvironment.
• ECM and matrisome remodeling may predict skeletal outcome.
• Osteocyte lacunar changes reveal early microarchitectural damage.
• Mechanical loading may support bone repair in selected patients.
• New imaging endpoints can quantify myeloma bone remineralization.

## 1 Introduction

Multiple myeloma is a plasma cell malignancy that evolves within the bone marrow and is clinically defined not only by hematologic progression but also by profound skeletal morbidity in most patients [1]. Osteolytic lesions, fractures, pain, spinal instability, and impaired quality of life remain major complications despite modern anti-myeloma therapy [1]. Classically, myeloma bone disease has been explained by an imbalance between osteoclast activation and osteoblast suppression (Fig. 1) [1]. This paradigm remains valid, but it should be expanded to include the following perspective: osteolytic lesions persist even after deep hematologic responses, suggesting that myeloma bone disease leaves a durable skeletal imprint that is not necessarily reversed by tumor reduction alone [2]. This interpretation is supported by clinical imaging studies showing that osteolytic manifestations frequently persist despite hematologic response, that lesion remineralization is partial and heterogeneous, and that the degree of local microenvironmental disruption may determine the capacity for bone repair [2], [3], [4], [5].

**Fig. 1:**
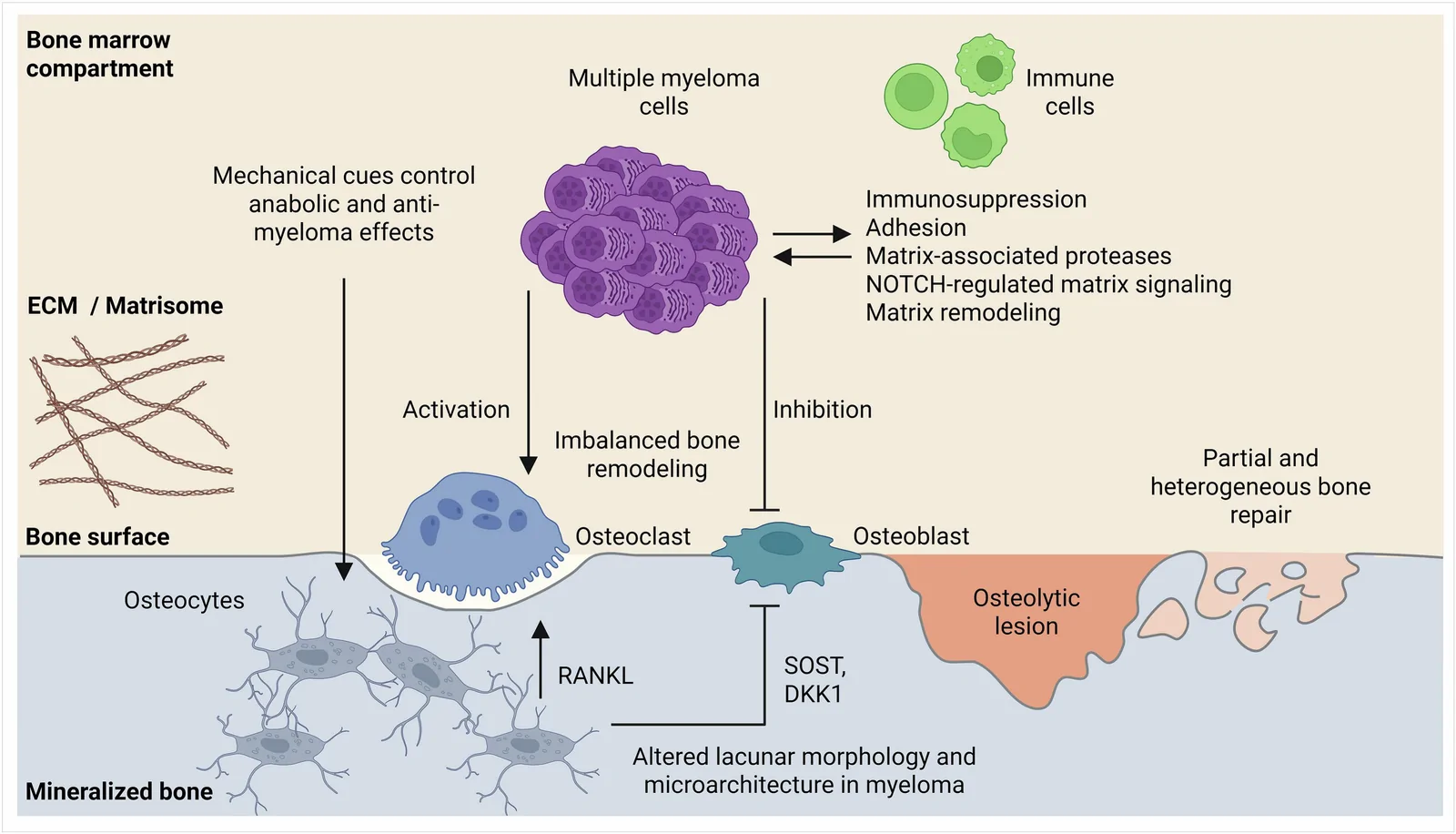
Schematic representation of the bone microenvironment in multiple myeloma. This diagram illustrates the complex interplay between malignant plasma cells, bone cells, immune cells, extracellular matrix (ECM) components, and mechanical cues within the bone marrow niche. Multiple myeloma cells promote osteoclast activation *via* Receptor Activator of Nuclear Factor Kappa-B Ligand (RANKL) while suppressing osteoblast function through factors such as sclerostin (SOST) and Dickkopf-1 (DKK-1), leading to an imbalance in bone remodeling and the formation of osteolytic lesions. In addition, immune cells within the microenvironment contribute to tumor progression and immune modulation through cytokine signaling and cell–cell interactions. The ECM and matrisome are depicted as active regulators of disease progression, providing structural support and signaling platforms that mediate adhesion, survival, and therapy resistance of myeloma cells. Matrix-associated proteases, together with NOTCH-driven matrisome programs, facilitate matrix remodeling and the release of growth factors. Osteocytes embedded in the mineralized matrix form a mechanosensitive lacuno-canalicular network integrating biochemical and mechanical signals. Despite therapeutic advances, bone repair remains incomplete and heterogeneous, with persistent osteolytic lesions. Mechanical loading and biophysical stimulation modulate bone turnover and control tumor growth. Overall, this model extends the classical osteoclast–osteoblast imbalance by incorporating immune regulation, ECM remodeling, osteocyte dysfunction, and mechanobiological influences as key drivers of myeloma bone disease.

Recent studies expand the field from a purely cell-centered model toward a microenvironmental and mechanobiological model of myeloma bone disease (Fig. 1). Importantly, this evolving concept increasingly integrates extracellular matrix (ECM) remodeling, vascular niche activation, and local immune dysregulation into a unified bone marrow ecosystem model (Fig. 1). Adhesion-dependent endothelial interactions and immune-protective microdomains appear to cooperate with osteolytic remodeling and stromal reprogramming to support tumor persistence, dissemination and impaired skeletal repair (Fig. 1). Osteocytes, extracellular matrix components, bone material properties, and mechanical cues are emerging as active regulators of skeletal destruction and repair [5], [6], [7], [8], [9]. This perspective is clinically relevant because antiresorptive treatment alone cannot rebuild lost bone, while tumor-directed therapy does not reliably restore skeletal integrity [1]. Sequential computer tomography (CT) studies demonstrate that remineralization can be quantified as a treatment endpoint, but also that repair remains incomplete and heterogeneous [2].

This review argues that novel insights into the bone microenvironment in myeloma bone disease converge on four principles: first, the ECM and matrisome, the collection of extracellular matrix proteins and ECM-associated molecules that regulate tissue structure and signaling, are not passive scaffolds but prognostically relevant and signaling-active structures; second, osteocytes and their lacuno-canalicular network represent a mechanosensitive interface between tumor, matrix, and bone remodeling; third, biophysical stimulation may become a rational adjuvant strategy to restore bone function, provided that patient selection and skeletal safety are rigorously addressed [4], [5], [6], [7], [8], [9], [10], [11]; and fourth, anti-myeloma therapies, including proteasome inhibitors, monoclonal antibodies, and chimeric antigen receptor (CAR) T-cell approaches, exert direct and indirect effects on bone remodeling, highlighting treatment-induced modulation of osteoimmune interactions as an emerging and still insufficiently understood determinant of skeletal disease evolution.

## 2 The extracellular matrix and matrisome as active components of the myeloma niche

The ECM is increasingly recognized as a dynamic regulator of myeloma progression rather than a passive architectural framework [12]. In the bone marrow, ECM proteins control adhesion, survival, migration, immune interactions, and therapy resistance [13]. Evers et al. identified recurrent mutations and altered expression of extracellular matrix gene families in multiple myeloma, including collagens, laminins, integrins, A Disintegrin And Metalloproteinase (ADAM)s, and ADAM with thrombospondin motifs (ADAMTS) family members [14]. In the Multiple Myeloma Research Foundation (MMRF) CoMMpass cohort, ECM gene mutations were frequent and associated with shorter progression-free survival, suggesting that matrix remodeling is biologically and clinically relevant [14].

This finding supports a broader view of myeloma biology: genetic and transcriptional alterations affecting adhesion and matrix composition may influence how plasma cells interact with stromal, endothelial, osteoblastic, osteoclastic, and immune compartments (Fig. 1). The ECM may therefore act as both a physical substrate and a signaling platform [13]. Importantly, ECM alterations may not simply reflect secondary tissue damage; they may help define biologically aggressive disease states [14].

Beyond these genetic and transcriptional alterations, ECM-associated proteases directly link matrix remodeling to oncogenic signaling pathways. Evers et al. demonstrated that high expression of the metalloproteinases ADAM8, ADAM9, and ADAM15 is associated with high-risk cytogenetic abnormalities, disease progression, and reduced survival in multiple myeloma patients [15]. Functional studies demonstrated that knockdown of these proteases attenuates proliferation-associated signaling, while mechanistically ADAM8 influences Insulin-Like Growth Factor 1 receptor (IGF1R)/protein kinase B (AKT) signaling and ADAM9 modulates mTOR activation [15]. These findings indicate that ECM-associated proteases actively regulate tumor cell signaling and contribute to disease progression beyond their structural role.

The matrisome concept further refines this argument. Maichl et al. showed that NOTCH signaling in myeloma cells regulates core matrisome and matrisome-associated genes [6]. NOTCH1 and NOTCH2 knockdown altered genes involved in cytokine signaling, ECM regulation, osteoclast differentiation, and bone remodeling, including Heparanase (HPSE), Matrix Metallopeptidase 13 (MMP13), S100 Calcium Binding Protein A6 (S100A6), Insulin-Like Growth Factor 1 (IGF1), Runt-Related Transcription Factor 2 (RUNX2), and Secreted Phosphoprotein 1 (SPP1) [6]. These findings connect oncogenic signaling with matrix remodeling and osteolytic biology. NOTCH-driven regulation of HPSE and MMP13 is highly relevant because heparanase and matrix metalloproteinases can modify proteoglycans, release matrix-bound factors, and support osteoclast-mediated bone degradation [6].

Together, these studies suggest that the myeloma matrisome may provide prognostic biomarkers and therapeutic targets. A key future direction is to determine whether ECM or matrisome signatures can predict skeletal complications, lesion persistence, or the capacity for remineralization after effective anti-myeloma treatment [6], [14].

A complementary layer of microenvironmental regulation is provided by mesenchymal stromal cell (MSC)-mediated retention and dissemination of myeloma cells. Kuric et al. developed an *in vitro* coculture model using primary human MSC and INA-6 myeloma cells to investigate how adhesion, proliferation, aggregation, and detachment shape early myeloma dissemination [16], with data analysis performed using a unified plotting and statistical workflow as previously described [17]. In this model, INA-6 cells first saturated the stromal cell surface and subsequently formed homotypic aggregates, from which single cells detached [16]. Importantly, detachment was linked to cell division: non-MSC-adherent daughter cells displayed a more proliferative phenotype, increased viability after IL-6 withdrawal, and upregulation of cell-cycle-associated genes, whereas MSC-adherent cells upregulated adhesion, extracellular matrix, and bone marrow retention factors including C-X-C motif chemokine ligand 12 (CXCL12), C-X-C motif chemokine ligand 8 (CXCL8), decorin (DCN), lysyl oxidase (LOX), transglutaminase 2 (TGM2), matrix metallopeptidase 2 (MMP2), and matrix metallopeptidase 14 (MMP14) [16].

This work refines the concept of the myeloma niche by showing that adhesion is biologically ambivalent. On one hand, stromal and ECM interactions retain malignant plasma cells within the bone marrow and may counteract dissemination; on the other hand, the same interactions provide survival and growth signals that support tumor persistence [16]. The functional consequences of myeloma cell adherence to the bone marrow microenvironment extend beyond physical retention within the niche. Macrophage inflammatory protein-1α (MIP-1α/CCL3), a chemokine highly expressed by myeloma cells, enhances tumor cell adhesion to marrow stromal cells through upregulation of β1-containing integrins, including α5β1. These adhesive interactions stimulate stromal production of pro-survival cytokines such as IL-6, thereby reinforcing tumor persistence and proliferation [18]. Simultaneously, MIP-1α promotes osteoclastogenesis by increasing Receptor Activator of Nuclear Factor Kappa-B Ligand (RANKL) expression in osteocytes and contributes to myeloma-associated bone destruction, highlighting how microenvironmental signaling pathways link skeletal deterioration to tumor progression [19].

Clinically, genes associated with the MSC-adherent phenotype were linked to longer progression-free and overall survival, whereas several retention-associated genes, including CXCL12, DCN, and TGM2, decreased during disease progression and relapse [16]. Thus, loss of bone marrow-retentive adhesion programs may represent a transition from niche dependence toward a more disseminated, microenvironment-independent disease state.

In this context, adhesion molecule-dependent vascular interactions emerge as a key determinant of dissemination. Brandl et al. identified junctional adhesion molecule-C (JAM-C) as a marker of a distinct CD138^low/neg^ myeloma cell population that preferentially localizes to perivascular niches and is associated with disease progression [20]. JAM-C expression inversely correlated with the canonical plasma cell marker CD138 and was enriched in myeloma cells interacting with bone marrow endothelium, suggesting a functional role in vascular engagement and dissemination [20]. Functionally, CD138^low/neg^ JAM-C^high^ cells displayed reduced proliferation but increased resistance to proteasome inhibition, consistent with a more therapy-resistant and dissemination-prone phenotype [20]. Importantly, therapeutic targeting of JAM-C *in vivo* reduced tumor burden and inhibited myeloma dissemination, highlighting adhesion-mediated endothelial interactions as a critical regulatory axis of disease progression [20].

Complementing these findings, junctional adhesion molecule-A (JAM-A) has emerged as another central regulator of the myeloma microenvironment. Solimando et al. demonstrated that JAM-A expression is increased in both myeloma plasma cells and bone marrow endothelial cells and correlates with adverse clinical outcome in newly diagnosed and relapsed/refractory multiple myeloma [21], [22]. Functionally, JAM-A promoted myeloma cell migration, proliferation, viability, and chemotaxis, while JAM-A blockade impaired tumor progression in preclinical *in vivo* models [21], [22].

Importantly, subsequent work identified endothelial JAM-A as a critical mediator of myeloma-associated angiogenesis [22]. Elevated JAM-A expression in myeloma endothelial cells enhanced angiogenic activity and supported tumor progression, whereas therapeutic JAM-A inhibition restored angiogenic homeostasis and reduced vascularization and tumor burden *in vivo* [22]. These findings position JAM-A at the interface of adhesion signaling, endothelial activation, vascular remodeling, and tumor dissemination.

Within the broader framework of myeloma bone disease, JAM-A supports the concept that the vascular niche is an active component of the diseased bone microenvironment rather than a passive transport compartment. Endothelial activation, angiogenesis, and adhesion-dependent vascular interactions may cooperate with ECM remodeling and immune suppression to establish microenvironmental conditions that favor myeloma persistence and skeletal destruction. Together with JAM-C-dependent vascular interactions, these findings support the concept that endothelial activation and adhesion signaling are integral functional components of the diseased myeloma bone niche rather than secondary bystander phenomena.

Taken together, these data integrate ECM composition, proteolytic remodeling, and adhesion-mediated niche interactions into a unified framework (Fig. 1). Matrix-associated signaling programs and adhesion dynamics are not only structural features of the myeloma microenvironment, but dynamic regulators of tumor localization, proliferation, survival, and dissemination (Fig. 1). These observations further suggest that ECM remodeling, endothelial activation, and immune organization are mechanistically interconnected components of the myeloma bone microenvironment.

Beyond ECM composition and adhesion signaling, the immune contexture of the bone marrow microenvironment has emerged as an important regulator of myeloma progression and persistence. The myeloma niche is not only structurally and mechanically remodeled, but also immunologically organized into local immune-protective microdomains that facilitate tumor survival. Increasing evidence suggests that immune dysregulation begins already at precursor stages such as monoclonal gammopathy of undetermined significance (MGUS) and evolves together with progressive remodeling of the bone marrow niche during transition to symptomatic multiple myeloma [23].

In this context, regulatory T-cells (Tregs) appear to play a central role. Dahlhoff et al. demonstrated in multiple myeloma patients and in syngeneic immunocompetent murine multiple myeloma models that CD4^+^Forkhead box (Fox)P3^+^ Tregs accumulate in close spatial proximity to malignant plasma cells within bone marrow compartments and display an activated phenotype [24]. Importantly, transient *in vivo* depletion of Tregs prevented myeloma dissemination and induced durable immune-mediated disease control through coordinated CD8^+^ T-cell and natural killer (NK)-cell responses [24]. These findings suggest that Tregs contribute to the establishment of an immune-protective niche that supports myeloma persistence and dissemination within the bone marrow. In parallel, additional suppressive immune populations, including myeloid-derived suppressor cells and dysfunctional NK/NKT-cell compartments, further contribute to local immune paresis within the myeloma niche [25], [26].

Recent spatial and single-cell analyses further indicate that the myeloma bone marrow is immunologically heterogeneous and organized into distinct regional immune states [27], [28]. Dhodapkar proposed a framework of spatial immune types in multiple myel, including immune-permissive, immune-excluded, immune-suppressed, immune-depleted, and immune-resistant lesions, emphasizing that local immune architecture may critically influence tumor persistence and therapeutic responsiveness [29]. Particularly relevant to bone disease biology is the observation that T-cell infiltration into myeloma lesions depends in part on local C-type lectin domain family 9 member a (Clec9a) positive dendritic cell networks, suggesting that regional immune organization within bone lesions directly regulates antitumor immunity [27]. Immune-excluded or immune-suppressed microdomains may therefore facilitate persistence of tumor cells within osteolytic niches despite systemic therapy.

Conceptually, these observations extend the current model of myeloma bone disease beyond osteoclast–osteoblast imbalance and ECM remodeling toward an integrated osteoimmunological framework. Immune cell composition, spatial immune organization, and local immune suppression may directly influence whether the bone microenvironment permits tumor persistence, dissemination, skeletal destruction, or tissue repair. Notably, progression from MGUS to multiple myeloma has been associated with loss of stem-like T-cell factor (TCF)1^+^ memory T-cells and accumulation of exhausted or terminally differentiated T-cell populations, supporting the concept of progressive immune aging within the marrow niche during myelomagenesis [30], [31].

The interaction between immune contexture and bone remodeling is particularly relevant because osteoclasts, stromal cells, endothelial cells, osteocytes, and inflammatory myeloid populations participate in cytokine and chemokine networks that regulate both bone turnover and immune-cell recruitment (Fig. 1). Moreover, stromal remodeling in multiple myeloma is increasingly recognized as inflammatory and immunoregulatory rather than purely structural [32]. Thus, immune-protective niches may represent an additional mechanism by which myeloma remodels the bone marrow ecosystem and maintains residual disease despite effective systemic therapy. These findings further support the emerging concept that spatial immune contexture should be considered as an integral functional component of the myeloma bone niche and may become increasingly relevant for future immunotherapeutic and bone-targeted treatment strategies.

## 3 Osteocytes as central regulators of myeloma-induced skeletal dysfunction

Osteocytes are ideally positioned to integrate mechanical, biochemical, and cellular signals in bone [33]. They regulate osteoclastogenesis through RANKL/osteoprotegerin (OPG) signaling and suppress osteoblast activity through sclerostin (SOST) and Dickkopf 1 (DKK-1) [33]. In myeloma bone disease, osteocytes are not merely bystanders. They are targets and mediators of disease-induced bone remodeling [7]. Recent clinical evidence has extended these preclinical observations by demonstrating that anti-sclerostin antibody treatment can promote repair of osteolytic lesions in a small cohort of patients with multiple myeloma in remission [34]. These findings support the therapeutic potential of targeting osteocyte-derived sclerostin to facilitate skeletal recovery following disease control.

Beyond mechanosensing, osteocytes participate in contact-dependent, paracrine, and extracellular-vesicle-mediated signaling that supports multiple myeloma progression [35], [36]. Delgado-Calle et al. demonstrated that direct osteocyte–myeloma interactions activate bidirectional NOTCH signaling, stimulating myeloma cell proliferation while initiating osteocyte apoptosis, which is further amplified by tumor-derived tumor necosis factor (TNF)α [37]. Subsequent work identified NOTCH3 as a mediator of osteocyte–myeloma communication that promotes tumor growth and bone destruction [38]. Separately, Anloague et al. identified a C-C motif chemokine ligand (CCL)3–high mobility group box (HMGB)1 pathway in which myeloma-derived CCL3 induces osteocytic HMGB1 release and RANKL expression independently of osteocyte apoptosis [19]. Thus, osteocyte dysfunction includes distinct cell-death-dependent and cell-death-independent responses to myeloma.

Osteocyte-associated soluble factors also connect the tumor niche to matrix remodeling and angiogenesis. Suvannasankha et al. showed that fibroblast growth factor (FGF)23, a hormone predominantly produced by osteocytes, is elevated and stimulates heparanase expression in myeloma cells through FGF receptor signaling [39]. FGFR inhibition reduced myeloma growth in an *ex vivo* bone model [39]. Mulcrone et al. demonstrated that hypoxia and myeloma-osteocyte interactions increase osteocytic VEGF-A production and that osteocyte-derived VEGF-A promotes endothelial tube formation. FGF23 further enhanced VEGF-A expression in osteocytes, linking their endocrine and angiogenic functions [40].

Osteocytes also contribute to therapy resistance. Sabol et al. showed that osteocyte-derived signals activate NOTCH3 in myeloma cells, inducing tumor-cell CXCL12 expression and downstream C-X-C motif chemokie receptor (CXCR)4 signaling that protects against bortezomib-based treatment [41]. Interruption of this pathway restored drug sensitivity in preclinical models [41]. In a distinct mechanism, Cheng et al. demonstrated that osteocyte-derived exosomes confer chemoresistance by promoting cancer stem cell-like properties through exosomal microRNA (miR-483)-3p and miR-513a-5p and hypoxia-inducible factor (HIF)-1α stabilization [42].

Moreno-Jimenez et al. provided important evidence by applying high-resolution microCT to human iliac crest biopsies from patients with monoclonal gammopathy of MGUS, smoldering multiple myeloma, and newly diagnosed multiple myeloma [7]. The study identified disease-associated changes in osteocyte lacunar morphology, including a trend toward lower lacunar density and larger lacunae with disease progression, as well as higher lacunar density and microporosity in larger lacunae in newly diagnosed myeloma compared with precursor conditions [7].

These observations are conceptually important because they move the field below the resolution of standard clinical imaging. Low-dose CT can detect osteolytic lesions [2], but microstructural changes in osteocyte lacunae may precede or accompany macroscopic bone destruction [7]. If validated in larger cohorts, osteocyte lacunar architecture could become a tissue-level biomarker of skeletal fragility, disease progression, or impaired repair capacity [7].

Mechanistically, altered lacunar morphology may reflect osteocyte apoptosis, perilacunar remodeling, osteocytic osteolysis, or changes in local matrix mineralization [7], [43]. The relevant question is no longer whether myeloma affects osteocytes, but whether altered osteocyte architecture actively contributes to a mechanically incompetent and poorly repairable bone niche [7].

## 4 Mechanobiology and experimental models of osteocyte function

Understanding osteocyte mechanobiology requires models that reproduce three-dimensional architecture, fluid flow, matrix strain, and cell-matrix interactions [8]. Conventional two-dimensional cultures cannot fully model the lacuno-canalicular environment [8]. Rindt et al. addressed this gap by developing a three-dimensional (3D) perfusion bioreactor model using osteocytic murine IDG-SW3 cells cultured on biomimetic scaffolds under fluid flow and compressive loading [8].

The model supported osteogenic differentiation, upregulated osteocytic markers including alkaline phosphatase (*Alpl*), dentin matrix protein 1 (*Dmp1*), and Sclerostin (*Sost*), and induced mechanoresponsive genes such as Fos proto-oncogene (*Fos*) and prostaglandin-endoperoxide synthase 2 (*Ptgs2*) under perfusion and cyclic compression [8]. These findings are supported by complementary 3D polyurethane scaffold-based systems demonstrating that fluid flow and compression reproducibly induce osteogenic differentiation, ECM formation, and activation of mechanotransduction pathways in human mesenchymal stromal cells [10]. Importantly, both platforms may be adapted to include osteoclasts, endothelial cells, stromal cells, or myeloma cells, thereby allowing analysis of tumor-bone crosstalk under controlled mechanical stimulation [8], [10]. The 3D models are particularly relevant for myeloma bone disease because they enable mechanistic dissection of how bone cells, and especially osteocytes, respond to physiologic or pathologic mechanical cues in a controlled environment [8], [10].

Such systems may bridge the gap between molecular studies and *in vivo* models, allowing researchers to test whether myeloma cells impair osteocytic mechanotransduction, whether anti-myeloma drugs restore it, and whether mechanical stimulation alters tumor-bone crosstalk.

## 5 The concept of biophysical stimulation as an anabolic and anti-myeloma stimulus

To study the effect of biophysical stimulation in the form of compressive mechanical loading, Rummler et al. used the syngeneic MOPC315.BM.Luc mouse model and intratibially injected myeloma cells [9]. In this model, controlled tibial mechanical loading prevented the progression of osteolytic destruction and improved cortical and trabecular bone microarchitecture [9]. Time-lapse microCT showed that loading altered bone remodeling at endosteal and periosteal surfaces, while bioluminescence imaging and serum myeloma protein measurements indicated reduced local tumor growth and dissemination [9]. These findings are highly significant because they suggest that biophysical stimulation may exert both bone-anabolic and tumor-modifying effects.

A later study by Rummler et al. extended this concept from bone quantity to bone quality [5]. In the same myeloma bone disease model, multiple myeloma-injected mice showed decreased cortical elastic modulus and hardness by nanoindentation, while cortical composition and mineral crystal properties were largely unchanged; cancellous bone mineral particles were thinner in multiple myeloma-injected mice [5]. Mechanical loading did not adversely alter bone composition, mineral structure, or mechanical properties in the context of myeloma [5]. The authors concluded that mechanically induced bone formation preserved structural and compositional integrity, supporting the potential of biophysical stimuli as an adjuvant anabolic therapy [5].

Building on these functional studies, Ziouti et al. provided a molecular framework for the response to mechanical loading in the same syngeneic MOPC315.BM model [11]. Transcriptomic profiling of an osteocyte-enriched cortical bone population revealed suppression of ECM organization and ossification-related programs after myeloma engraftment [11]. A single tibial loading session counterregulated part of this transcriptional response [11]. Comparison with the Naba matrisome [44] identified 94 loading-responsive ECM-associated genes [11]. Cross-species analysis linked eight of these genes to overall survival in a cohort of 387 patients, and a three-dimensional IDG-SW3 bioreactor confirmed load responsiveness of *Vegfa*, *Ccl2*, *Sdc1*, and *Sema3a* [11]. Together, these results support the reactivation of mechanically responsive matrix and repair programs as a potential contributor to the skeletal effects reported in the earlier loading studies [5], [9]. How these loading-responsive programs interact with osteocyte-mediated tumor-supportive signaling remains unresolved. Their causal contribution to the previously observed reduction in tumor burden, and the relative roles of osteocytes, other skeletal cells, and vascular or immune compartments, require further study [11].

## 6 Translational perspective of biophysical stimulation and challenges for skeletal repair in patients with myeloma

To transfer the knowledge about anabolic effects and bone structural changes after biophysical stimuli in preclinical models, two pilot clinical studies were conducted [2], [4]. In a study by Grunz et al. a reliable endpoint for assessing bone remineralization in newly diagnosed multiple myeloma was established by performing sequential whole-body low-dose CT [2]. In 20 patients treated with elotuzumab, carfilzomib, lenalidomide and dexamethosone induction therapy, osteolytic lesion size decreased after six cycles, inter-reader reliability was high, and trabecular sclerosis was strongly associated with lesion-level response [2]. This study is important because it operationalizes bone repair as a measurable endpoint rather than a descriptive radiologic impression. It also indicates that modern induction therapy may permit partial structural recovery, particularly where trabecular sclerosis develops [2].

Kollikowski et al. translated the concept of biophysical stimulation into a prospective pilot study of impact-loading exercise in patients with multiple myeloma [4]. After careful selection, including exclusion of patients with recent fractures or spinal instability and assessment using the spinal instability neoplastic score, impact-loading exercise appeared feasible and safe in selected patients [4]. However, no CT signs of bone remineralization were observed after six months, despite improvements in selected physical performance and quality-of-life measures [4].

While Grunz et al. show that remineralization can occur and can be measured under effective systemic therapy [2], Kollikowski et al. show that exercise can be safely implemented but may not produce detectable structural repair over six months in a small heterogeneous cohort [4]. The translational challenge is therefore not only to apply biophysical stimulation, but to define the therapeutic window, skeletal eligibility criteria, lesion types most likely to respond, and imaging endpoints sensitive enough to detect early repair. In addition, future translational strategies may need to account for the biological heterogeneity of the bone marrow niche itself. Regional immune suppression, endothelial activation, and adhesion-dependent vascular niches may influence whether skeletal lesions retain the capacity for effective remineralization or remain sites of persistent residual disease despite systemic therapy [22], [24], [29].

## 7 Impact of myeloma therapies on bone metabolism

Since biophysical stimulation has not yet reached clinical application, patients with myeloma bone disease suffering from skeletal-related events (SREs) and having a diminished quality of life largely receive standard bone protective treatment such as zoledronic acid and denosumab, in which the latter prevents the stronger occurrence of SREs [45], [46], [47], [48]. In addition, anti-myeloma medications themselves affect the bone metabolism (Fig. 2).

**Fig. 2:**
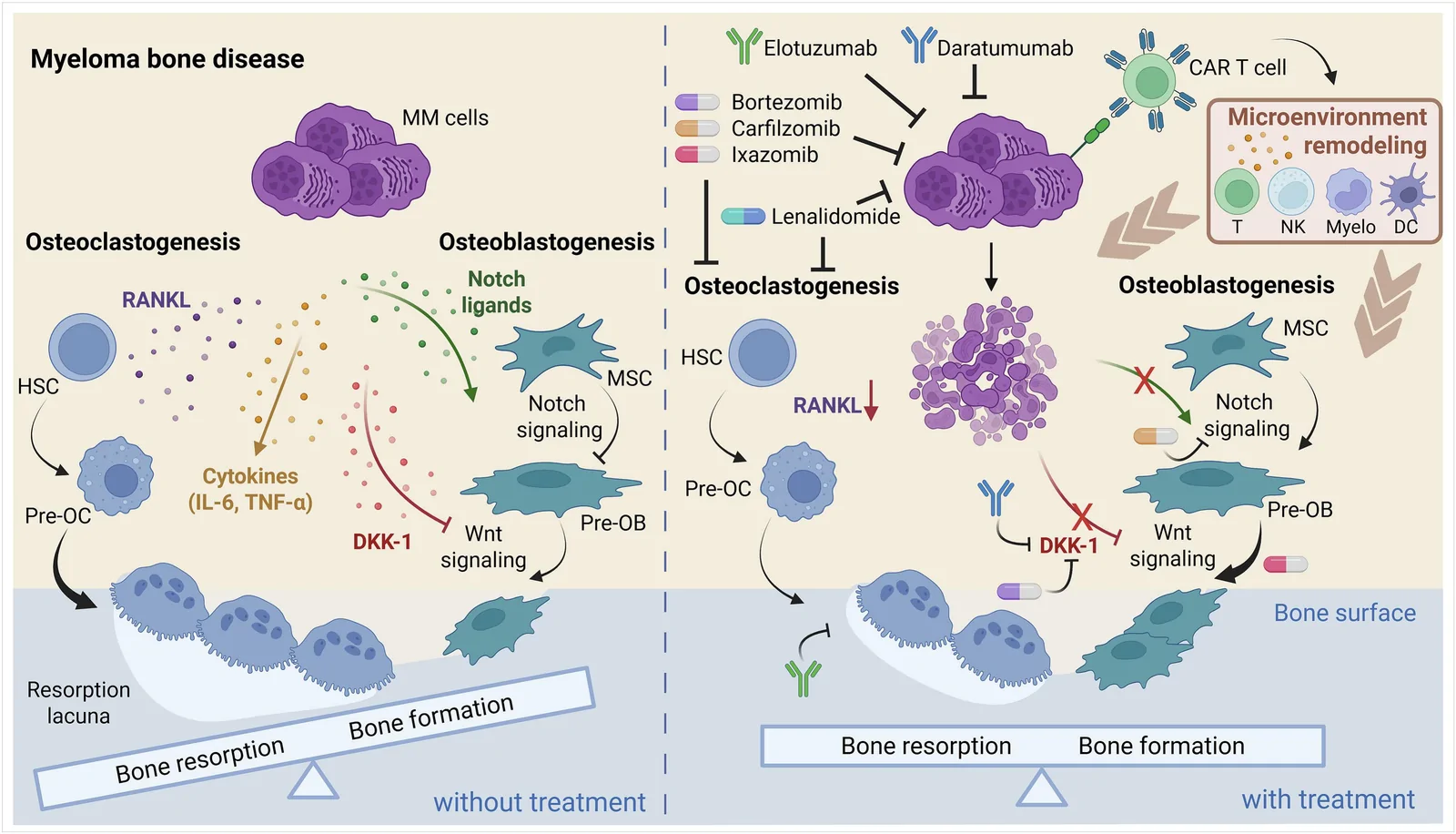
Schematic overview of the impact of multiple myeloma therapies on bone homeostasis. Multiple myeloma (MM) cells disrupt bone homeostasis by regulating pathways in bone-forming osteoblasts. They secrete elevated levels of NOTCH ligands and Dickkopf-1 (DKK-1), resulting in enhanced NOTCH signaling and inhibition of Wnt signaling, thereby suppressing osteoblast differentiation and bone formation. Simultaneously, MM cells stimulate the expression of Receptor Activator of Nuclear Factor Kappa-B Ligand (RANKL) in osteoblasts and their increased release of pro-inflammatory cytokines promote osteoclast activity. The resulting imbalance between impaired bone formation and increased bone resorption drives the development of osteolytic bone lesions characteristic of multiple myeloma (left). Therapeutic strategies for patients with MM include monoclonal antibodies (daratumumab, elotuzumab), proteasome inhibitors (bortezomib, carfilzomib, ixazomib), and immunomodulatory drugs (lenalidomide), or chimeric antigen receptor (CAR) T-cell therapy, which promote apoptosis of MM cells leading to the remodeling of the myeloma bone niche. The reduction in MM cell burden decreases the production of NOTCH ligands and DKK-1, resulting in attenuation of NOTCH signaling and activation of Wnt signaling, which enhance bone formation. In addition to inducing MM cell death, CAR T-cells may further modulate other hematopoietic cells. Among proteasome inhibitors, ixazomib directly stimulates bone formation. Furthermore, proteasome inhibitors, lenalidomide, and elotuzumab suppress osteoclast activity, thereby reducing bone resorption. Overall, treatment-induced remodeling of the myeloma bone niche contributes to the restoration of bone formation and resorption balance and limit the development of new osteolytic lesions (right).

Bortezomib is employed as a first-generation proteasome inhibitor, which leads to the toxic accumulation of proteins, thereby triggering cell death in myeloma cells. Concurrently, it offers benefits for osteogenesis (Fig. 2). Plasma cells produce elevated levels of DKK-1, whose inhibition by bortezomib promotes Wnt-mediated osteogenic differentiation of MSC from patients with myeloma *in vitro* [49], [50]. Even patients with smoldering multiple myeloma benefit from low-dose bortezomib by increased bone mineral density [51]. Whereas higher bortezomib doses are administered either as first-line therapy or in the setting of relapsed multiple myeloma. Relapsed patients display significantly higher serum levels of DKK-1 and sRANKL, together with an elevated RANKL/OPG ratio compared to healthy controls, reflecting enhanced osteoclastogenic activation and the presence of osteolytic lesions. Treatment with four cycles of bortezomib reduces these proteins. Nevertheless, existing osteolytic lesions fail to undergo healing [52]. The concomitant administration of dexamethasone further stimulates osteoblast formation and prevents the development of new osteolytic lesions, while pre-existing lesions persist [53]. Furthermore, adding lenalidomide to the bortezomib/dexamethasone regimen in patients with multiple myeloma highlights the importance of bortezomib in activating bone formation, as treatment without bortezomib merely reduces bone resorption [54]. These findings provide a rationale for using bortezomib as a consolidation therapy following autologous stem cell transplantation. Although bortezomib does not alter bone mineral density compared to untreated patients, it is associated with prolonged progression-free survival [55]. In combination with lenalidomide, the therapy decreases the RANKL/OPG ratio and prevents the occurrence of new SREs [56]. By contrast, consolidation with thalidomide and dexamethasone results in a low incidence of SRE despite continued suppression of osteoclast activity, highlighting potential differences in the effect of consolidation regimens on the bone homeostasis [57].

Similar to bortezomib, the second-generation proteasome inhibitor carfilzomib also exerts effects on bone metabolism in myeloma (Fig. 2). In contrast to the DKK-1-mediated inhibition of Wnt signaling, NOTCH signaling is constitutively activated in MSCs from patients with multiple myeloma. Carfilzomib blocks NOTCH1, thereby promoting osteoblast activation, differentiation and mineralization. Simultaneously, the treatment suppresses osteoclast-mediated bone resorption, leading to an overall anabolic effect on bone and contributing to the prevention of SREs [58], [59], [60], [61]. However, administered as consolidation therapy in combination with lenalidomide/dexamethasone, carfilzomib merely restricts osteoclast activity [62].

Finally, there is a third-generation proteasome inhibitor: ixazomib, which has been reported to exert favorable effects on bone of patients with multiple myeloma as well (Fig. 2). A preclinical study demonstrated that ixazomib suppresses plasma cell-induced osteoclastogenesis and indirectly enhances osteoblast differentiation *via* Sonic Hedgehog (SHH) signaling [63]. Clinically, 3-month medication with ixazomib resulted in increased bone volume due to inhibition of osteoclast activity, with no significant changes in the osteoblast function [64]. Furthermore, patients in remission appear to derive sustained skeletal benefit from ixazomib therapy, as the treatment has been linked to prolonged bone formation and improved bone mineralization over a period of two years [65].

Currently, the therapeutic options for patients with multiple myeloma are extended to include monoclonal antibodies. Daratumumab, a monoclonal antibody targeting CD38, which is highly expressed on malignant plasma cells, is widely used in both newly diagnosed and relapsed/refractory patients with multiple myeloma. By inducing plasma cell apoptosis, daratumumab reduces circulating levels of DKK-1, thereby alleviating Wnt signaling inhibition and promoting bone formation [66]. However, it does not simultaneously reduce the incidence of SREs [67]. In combination regimens comprising daratumumab, bortezomib, lenalidomide, and dexamethasone, followed by lenalidomide consolidation after autologous stem cell transplantation, daratumumab has been associated with prolonged progression-free survival [68]. Furthermore, the use of thalidomide instead of lenalidomide has shown improved survival as well compared with corresponding treatment regimens without daratumumab [69], [70]. Nevertheless, the impact of these therapeutic approaches on bone remodeling and skeletal integrity remains insufficiently characterized.

Another monoclonal antibody used in multiple myeloma is elotuzumab, which targets Signaling Lymphocytic Activation Molecule Family Member 7 (SLAMF7). Elotuzumab enhances NK cell-mediated cytotoxicity, promoting apoptosis of malignant plasma cells [71]. Since SLAMF7 is also expressed on osteoclasts, it suggests a direct effect on bone resorption through osteoclast depletion [72]. Consistent with this mechanism, treatment with elotuzumab in combination with carfilzomib, lenalidomide, and dexamethasone has been associated with a reduction in osteolytic lesions after six treatment cycles [2].

In the last years, B-Cell Maturation Antigen (BCMA)-directed chimeric antigen receptor (CAR) T-cell therapies have fundamentally changed the treatment landscape of relapsed/refractory multiple myeloma, achieving deep and durable remissions in a substantial proportion of patients [73]. While clinical studies and translational research have primarily focused on tumor control, cytokine release syndrome (CRS), neurotoxicity and hematologic recovery, the effects of CAR T-cell therapy on the bone microenvironment remain largely unexplored [73].

First preclinical studies revealed that tumor control by conventional BCMA-CAR T-cells alone may be insufficient to prevent or even reverse myeloma-associated bone destruction [74]. Sun et al. developed a fourth-generation BCMA-cystatin E/M (CST6)-CAR construct designed to inhibit osteoclast differentiation and function in a CST6-dependent manner [74]. In addition to efficient elimination of myeloma cells, BCMA-CST6-CAR-T-cells reduced the number of Tartrate-Resistant Acid Phosphatase (TRAP)-positive osteoclasts and prevented osteolytic lesions in a xenograft mouse model [74]. These findings indicated that, similar to observations made with proteosome inhibitors, effective tumor control alone does not necessarily result in normalization of bone remodeling [74]. As one of the first clinical datasets addressing this question, Ussmann et al. longitudinally assessed bone turnover markers following BCMA-directed CAR-T-cell therapy [75]. While bone resorption appeared to be transiently increased shortly after CAR T-cell infusion, potentially associated with CRS-related inflammation, clinical response to therapy was accompanied by measurable alterations in bone turnover markers after resolution of the inflammatory phase [75]. These observations suggest that skeletal remodeling responds dynamically to CAR-T-cell-induced tumor eradication [75].

In contrast to proteasome inhibitors and immunomodulatory imide drugs (IMiDs), which exert relatively direct effects on osteoclasts, osteoblasts and their associated signaling pathways, CAR-T-cell therapy additionally remodels the immune and bone marrow microenvironment and may therefore exert indirect effects on the skeletal system (Fig. 2). This concept is consistent with the current understanding that myeloma bone disease arises from a complex osteoimmunological network within the bone marrow microenvironment [76], [77], [78]. In this context, imaging studies by Born et al. demonstrated profound changes in the spatial distribution and metabolic activity of myeloma lesions following CAR T-cell therapy, potentially providing a framework for future investigations of skeletal repair after treatment [79]. Furthermore, dynamic alterations of immune cell populations following CAR T-cell therapy may have implications beyond tumor control [80], as many of these cellular subsets are established regulators of osteoclastogenesis and bone remodeling including Tregs and other non-CAR T-cell populations. These observations are supported by comprehensive single-cell analyses of the bone marrow microenvironment following BCMA-directed CAR T-cell therapy. Both the multiomic single-cell study and the longitudinal single-cell atlas by Rade et al. demonstrated profound remodeling of the bone marrow ecosystem during response and resistance to CAR T-cell therapy [81], [82]. Altered cellular populations include myeloid cells, dendritic cells, and diverse immune cell subsets, accompanied by extensive changes in inflammatory signaling pathways and cellular interaction networks [81], [82]. Notably, many of these cellular compartments and signaling axes are also recognized contributors to the pathogenesis of myeloma bone disease (Fig. 2). Given the intimate crosstalk between immune cells, stromal cells, osteoclasts and osteoblasts within the myeloma niche, these findings raise the possibility that CAR T-cell therapy may affect bone remodeling not only through elimination of malignant plasma cells, but also through reprogramming of osteoimmune interactions (Fig. 2). Taken together, emerging clinical observations of altered bone turnover markers after CAR T-cell therapy and recent single-cell studies demonstrating extensive remodeling of the bone marrow ecosystem provide a first rationale for investigating CAR T-cell-induced changes in osteoimmune homeostasis as a previously underappreciated component of treatment response.

While currently approved BCMA-directed CAR T-cell products primarily act through elimination of malignant plasma cells, next-generation engineered CAR T-cells may actively remodel the bone marrow microenvironment [83]. Fourth-generation “armored” CAR-T-cells capable of secreting cytokines or checkpoint inhibitors, such as IL12-secreting or anti-PD-1/anti-PD-L1-secreting CAR-T-cells, may simultaneously reduce tumor burden and modulate myeloma bone disease through immunomodulatory effects on the tumor niche [74], [83]. In the future, CAR T-cell constructs engineered to secrete bone-modifying factors, such as OPG, anti-DKK-1 antibodies or anti-sclerostin molecules, may become conceivable strategies to directly target key drivers of myeloma bone disease. Such approaches may ultimately transform CAR T-cell therapy from purely anti-myeloma strategy into a platform capable of restoring osteoimmune homeostasis within the bone marrow niche.

## 8 Critical synthesis

The reviewed studies support a new model of myeloma bone disease in which the bone microenvironment is altered at molecular, cellular, structural, and mechanical levels. ECM remodeling and adhesion-dependent programs emerge as central regulators linking tumor-microenvironment interactions to proliferation, therapy resistance, and dissemination. MIP-1α-dependent upregulation of α5β1 integrin-mediated adhesion further illustrates how stromal interactions can simultaneously enhance tumor survival, cytokine support, and osteoclast activation, thereby coupling niche retention to disease progression and bone destruction [19]. ECM mutations and NOTCH-driven matrisome programs suggest that matrix composition and matrix-associated signaling are linked to myeloma progression and patient survival [6], [14]. This concept is further extended by *in vitro* work demonstrating that stromal adhesion and ECM-associated gene programs regulate the balance between bone marrow retention and dissemination, suggesting that myeloma progression may involve a shift from microenvironmental anchorage toward increased niche independence [16]. Adhesion-mediated vascular interactions, including JAM-C- and JAM-A-dependent endothelial signaling, reinforce the notion that dissemination is an actively regulated process within the bone marrow niche rather than passive hematogenous spread [20]. JAM-A additionally links endothelial activation and angiogenesis to microenvironmental remodeling and adverse clinical outcome, emphasizing the functional importance of the vascular niche in myeloma progression [21], [22]. The emerging immune-contexture data further expand this framework toward an osteoimmunological model of myeloma bone disease. Spatial immune suppression, Treg accumulation, dysfunctional NK/T-cell compartments, and inflammatory stromal remodeling suggest that osteolytic lesions may function as locally immune-protective microdomains that support tumor persistence despite systemic therapy [24], [27], [29]. At the structural level, osteocyte lacunar changes in human MGUS, smoldering multiple myeloma, and multiple myeloma biopsies indicate progressive disruption of bone microarchitecture during disease evolution [7]. Moreover, accumulating evidence suggests that osteocytes actively participate in myeloma progression through bidirectional signaling with malignant plasma cells, extending their role beyond mechanosensation and skeletal maintenance toward active regulation of the tumor microenvironment [42]. Consistently, preclinical loading studies demonstrate that bone remains mechanically responsive even in the presence of myeloma [5], [9], while clinical studies show that both CT-based remineralization endpoints and exercise interventions are feasible [2], [4].

However, several gaps remain. First, most available human studies are small and observational or pilot in nature [2], [4], [7]. Second, biopsy-based microCT provides exquisite resolution but is limited by sampling site, tissue availability, and uncertainty as to whether iliac crest biopsies represent focal lesions or systemic bone alterations [7]. Third, the relationship between osteocyte lacunar morphology, molecular osteocyte dysfunction, and clinical fracture risk remains deductive [7], [8]. Longitudinal studies integrating imaging, serum bone turnover markers, bone biopsies, and spatial molecular profiling will be necessary to establish temporal and functional relationships.

## 9 Conclusion

Novel insights into the bone microenvironment in myeloma bone disease reveal a complex and dynamic niche in which tumor cells, osteoclasts, osteoblasts, osteocytes, ECM components, endothelial structures, immune compartments, and mechanical cues interact. The classical osteoclast-osteoblast imbalance remains central, but it should be embedded within a broader framework that includes matrisome remodeling, osteocyte dysfunction, osteocyte-mediated tumor-supportive signaling, lacunar microarchitecture, bone material properties, and mechanotransduction [2], [4], [5], [6], [7], [8], [9], [14]. Emerging evidence indicates that immune contexture and adhesion-dependent vascular remodeling are also integral functional components of this diseased ecosystem and may contribute to dissemination, angiogenesis, immune escape, and persistent skeletal lesions despite hematologic response. This expanded model has direct clinical implications. It suggests that future skeletal therapy in myeloma should not only inhibit resorption but also restore bone formation, matrix integrity, osteocyte function, and mechanical competence. CT-based lesion measurement, high-resolution bone microarchitecture, and mechanobiological biomarkers may together define a new generation of endpoints for skeletal response [2], [4], [7], [8].

## 10 Future perspectives

Future research should prioritize five directions. First, ECM and matrisome signatures should be validated as predictors of skeletal events, progression-free survival, and treatment response [6], [14]. Second, osteocyte lacunar morphology should be studied in larger MGUS, smoldering multiple myeloma, and multiple myeloma cohorts, ideally with spatial correlation to focal lesions and fracture risk [7]. Third, three-dimensional bioreactor models should be used to dissect how myeloma cells alter osteocyte mechanotransduction and whether therapeutic agents can restore it [8], [10]. Fourth, mechanical loading should be tested in rationally designed clinical trials with strict skeletal safety screening, lesion-specific imaging endpoints, and longer follow-up [6], [7], [8], [10]. Fifth, multimodal approaches combining anti-myeloma therapy, antiresorptives, anabolic or osteocyte-targeted agents, vascular or immune microenvironment-directed therapies, and supervised mechanical stimulation should be investigated. Spatial immune profiling, vascular niche characterization, and integration of osteoimmunological biomarkers may further help identify patients with persistent immune-protective or dissemination-permissive bone marrow microenvironments. The field is moving toward a central translational hypothesis: skeletal repair in myeloma will require not only eradication or suppression of malignant plasma cells, but active reprogramming of the diseased bone microenvironment. This concept may define the next phase of research in myeloma bone disease and provides a strong rationale for integrating bone biology, mechanobiology, imaging, and immunooncology into future therapeutic strategies.

## Graphic software

The figures were created in BioRender. Ebert, R. (2026) https://BioRender.com/cfvaypq and https://BioRender.com/3sqpc4t.

## Declaration of generative AI and AI-assisted technologies in the manuscript preparation process

During the preparation of this work the authors used ChatGPT (OpenAI; Codex, based on GPT 6) to assist with language editing and drafting. After using this tool, the authors reviewed and edited the content as needed and take full responsibility for the content of the published article*.*

## CRediT authorship contribution statement

**Franziska Jundt:** Writing – review & editing, Writing – original draft, Funding acquisition, Conceptualization. **Wen-Hui Cheng:** Writing – review & editing. **Poonam Kumari:** Writing – review & editing. **Heike Weidner:** Writing – review & editing, Writing – original draft, Funding acquisition, Conceptualization. **Luise Fischer:** Writing – review & editing, Writing – original draft. **Maximilian Merz:** Writing – review & editing, Funding acquisition. **Andreas Beilhack:** Writing – review & editing, Writing – original draft, Funding acquisition. **Martin Kuric:** Writing – review & editing. **Regina Ebert:** Writing – review & editing, Writing – original draft, Funding acquisition, Conceptualization.

## Funding sources

This work was funded by the Deutsche Forschungsgemeinschaft (DFG, German Research Foundation) within the Priority Program SPP 2084 μBONE, Project numbers 491715122 (F.J. and R.E.), 491584344 (H.W. and M.M.), and 401253051 (A.B.), and by the DFG, project numbers 496963451 (F.J) and 530200354 (F.J and R.E.).

## Declaration of competing interest

The authors declare that they have no known competing financial interests or personal relationships that could have appeared to influence the work reported in this paper.

## References

[1] Swan D., Yong A., Vandyke K., Hocking J. (2026). Myeloma bone disease: a constant problem in the changing landscape of myeloma management. Br. J. Haematol..

[2] Grunz J.P., Kunz A.S., Baumann F.T., Hasenclever D., Sieren M.M., Heldmann S., Bley T.A., Einsele H., Knop S., Jundt F. (2023). Assessing osteolytic lesion size on sequential CT scans is a reliable study endpoint for bone remineralization in newly diagnosed multiple myeloma. Cancers (Basel).

[3] Hinge M., Andersen K.T., Lund T., Jorgensen H.B., Holdgaard P.C., Ormstrup T.E., Ostergaard L.L., Plesner T. (2016). Bone healing in multiple myeloma: a prospective evaluation of the impact of first-line anti-myeloma treatment. Haematologica.

[4] Kollikowski A., Schallock M., Ringeisen R., Hasenclever D., Seefried L., Grunz J.P., Zubac D., Loffler C., Baumann F.T., Jundt F. (2025). Feasibility and safety of impact-loading exercise in patients with multiple myeloma-a pilot study. Support Care Cancer.

[5] Rummler M., Ziouti F., Snyder L., Zimmermann E.A., Lynch M., Donnelly E., Wagermaier W., Jundt F., Willie B.M. (2025). Bone mechanical properties were altered in a mouse model of multiple myeloma bone disease. Biomater. Adv..

[6] Maichl D.S., Kirner J.A., Beck S., Cheng W.H., Krug M., Kuric M., Ade C.P., Bischler T., Jakob F., Hose D., Seckinger A., Ebert R., Jundt F. (2023). Identification of NOTCH-driven matrisome-associated genes as prognostic indicators of multiple myeloma patient survival. Blood Cancer J..

[7] Moreno-Jimenez I., Heinig S., Heras U., Maichl D.S., Strifler S., Leich E., Blouin S., Fratzl P., Fratzl-Zelman N., Jundt F., Cipitria A. (2024). 3D osteocyte lacunar morphometry of human bone biopsies with high resolution microCT: from monoclonal gammopathy to newly diagnosed multiple myeloma. Bone.

[8] Rindt W.D., Krug M., Yamada S., Sennefelder F., Belz L., Cheng W.-H., Azeem M., Kuric M., Evers M., Leich E., Hartmann T.N., Pereira A.R., Hermann M., Hansmann J., Mussoni C., Stahlhut P., Ahmadi T., Yassin M.A., Mustafa K., Ebert R., Jundt F. (2024). A 3D bioreactor model to study osteocyte differentiation and mechanobiology under perfusion and compressive mechanical loading. Acta Biomater..

[9] Rummler M., Ziouti F., Bouchard A.L., Brandl A., Duda G.N., Bogen B., Beilhack A., Lynch M.E., Jundt F., Willie B.M. (2021). Mechanical loading prevents bone destruction and exerts anti-tumor effects in the MOPC315.BM.Luc model of myeloma bone disease. Acta Biomater..

[10] Boehm T.M., Müller-Deubert S., Krug M., Mussoni C., Stahlhut P., Stepanenko V., Rudert M., Kusan B., Docheva D., Ahmad T., Ebert R. (2026). 3D polyurethane scaffolds for exploring osteogenic differentiation and mechanical stimulation of mesenchymal stromal cells. Bone.

[11] Ziouti F., Azeem M., Rummler M., Rosolowski M., Zapata G., Rindt W., Julien C., Tauer J.T., Cheng W.H., Beck S., Seckinger A., Hose D., Leich E., Bogen B., Lynch M.E., Kuric M., Krug M., Ebert R., Zaucke F., Jakob F., Willie B.M., Jundt F. (2026). Myeloma engraftment suppresses osteocytic ossification signatures rescued by loading in mice and reveals predictors of patient outcome. Bone.

[12] Iannozzi N.T., Giuliani N., Storti P. (2025). Deciphering the bone marrow microenvironment's role in multiple myeloma immunotherapy resistance. Front. Immunol..

[13] Sleeboom J.J.F., van Tienderen G.S., Schenke-Layland K., van der Laan L.J.W., Khalil A.A., Verstegen M.M.A. (2024). The extracellular matrix as hallmark of cancer and metastasis: from biomechanics to therapeutic targets. Sci. Transl. Med..

[14] Evers M., Schreder M., Stuhmer T., Jundt F., Ebert R., Hartmann T.N., Altenbuchinger M., Rudelius M., Kuric M., Rindt W.D., Steinbrunn T., Langer C., Heredia-Guerrero S.C., Einsele H., Bargou R.C., Rosenwald A., Leich E. (2023). Prognostic value of extracellular matrix gene mutations and expression in multiple myeloma. Blood Cancer J..

[15] Evers M., Stuhmer T., Schreder M., Steinbrunn T., Rudelius M., Jundt F., Ebert R., Hartmann T.N., Bargou R.C., Rosenwald A., Leich E. (2024). Association of ADAM family members with proliferation signaling and disease progression in multiple myeloma. Blood Cancer J..

[16] Kuric M., Beck S., Schneider D., Rindt W., Evers M., Meissner-Weigl J., Zeck S., Krug M., Herrmann M., Hartmann T.N., Leich E., Rudert M., Docheva D., Seckinger A., Hose D., Jundt F., Ebert R. (2024). Modeling myeloma dissemination in vitro with hMSC-interacting subpopulations of INA-6 cells and their aggregation/detachment dynamics. Cancer Res. Commun..

[17] Kuric M., Ebert R. (2024). Plotastic: bridging plotting and statistics in Python. J. Open Source Softw..

[18] Oba Y., Lee J.W., Ehrlich L.A., Chung H.Y., Jelinek D.F., Callander N.S., Horuk R., Choi S.J., Roodman G.D. (2005). MIP-1alpha utilizes both CCR1 and CCR5 to induce osteoclast formation and increase adhesion of myeloma cells to marrow stromal cells. Exp. Hematol..

[19] Anloague A., Sabol H.M., Kaur J., Khan S., Ashby C., Schinke C., Barnes C.L., Alturkmani F., Ambrogini E., Gundesen M.T., Lund T., Amstrup A.K., Andersen T.L., Diaz-delCastillo M., Roodman G.D., Bellido T., Delgado-Calle J. (2025). A novel CCL3-HMGB1 signaling axis regulating osteocyte RANKL expression in multiple myeloma. Haematologica.

[20] Brandl A., Solimando A.G., Mokhtari Z., Tabares P., Medler J., Manz H., Da Via M.C., Croci G.A., Kurzwart M., Thusek S., Schneider T., Ebert R., Jakob F., Einsele H., Beilhack A. (2022). Junctional adhesion molecule C expression specifies a CD138low/neg multiple myeloma cell population in mice and humans. Blood Adv..

[21] Solimando A.G., Brandl A., Mattenheimer K., Graf C., Ritz M., Ruckdeschel A., Stuhmer T., Mokhtari Z., Rudelius M., Dotterweich J., Bittrich M., Desantis V., Ebert R., Trerotoli P., Frassanito M.A., Rosenwald A., Vacca A., Einsele H., Jakob F., Beilhack A. (2018). JAM-A as a prognostic factor and new therapeutic target in multiple myeloma. Leukemia.

[22] Solimando A.G., Da Via M.C., Leone P., Borrelli P., Croci G.A., Tabares P., Brandl A., Di Lernia G., Bianchi F.P., Tafuri S., Steinbrunn T., Balduini A., Melaccio A., De Summa S., Argentiero A., Rauert-Wunderlich H., Frassanito M.A., Ditonno P., Henke E., Klapper W., Ria R., Terragna C., Rasche L., Rosenwald A., Kortum M.K., Cavo M., Ribatti D., Racanelli V., Einsele H., Vacca A., Beilhack A. (2021). Halting the vicious cycle within the multiple myeloma ecosystem: blocking JAM-A on bone marrow endothelial cells restores angiogenic homeostasis and suppresses tumor progression. Haematologica.

[23] Dhodapkar M.V., Paiva B. (2025). Immune alterations in myeloma evolution and outcomes: quo vadis?. Blood.

[24] Dahlhoff J., Manz H., Steinfatt T., Delgado-Tascon J., Seebacher E., Schneider T., Wilnit A., Mokhtari Z., Tabares P., Bockle D., Rasche L., Martin Kortum K., Lutz M.B., Einsele H., Brandl A., Beilhack A. (2022). Transient regulatory T-cell targeting triggers immune control of multiple myeloma and prevents disease progression. Leukemia.

[25] Gorgun G.T., Whitehill G., Anderson J.L., Hideshima T., Maguire C., Laubach J., Raje N., Munshi N.C., Richardson P.G., Anderson K.C. (2013). Tumor-promoting immune-suppressive myeloid-derived suppressor cells in the multiple myeloma microenvironment in humans. Blood.

[26] Ramachandran I.R., Martner A., Pisklakova A., Condamine T., Chase T., Vogl T., Roth J., Gabrilovich D., Nefedova Y. (2013). Myeloid-derived suppressor cells regulate growth of multiple myeloma by inhibiting T cells in bone marrow. J. Immunol..

[27] Robinson M.H., Villa N.Y., Jaye D.L., Nooka A.K., Duffy A., McCachren S.S., Manalo J., Switchenko J.M., Barnes S., Potdar S., Azeem M.I., Horvat A.A., Parihar V.C., Gong J., Liang Y., Smith G.H., Gupta V.A., Boise L.H., Kaufman J.L., Hofmeister C.C., Joseph N.S., Lonial S., Dhodapkar K.M., Dhodapkar M.V. (2023). Regulation of antigen-specific T cell infiltration and spatial architecture in multiple myeloma and premalignancy. J. Clin. Invest..

[28] John L., Poos A.M., Brobeil A., Schinke C., Huhn S., Prokoph N., Lutz R., Wagner B., Zangari M., Tirier S.M., Mallm J.P., Schumacher S., Vonficht D., Sole-Boldo L., Quick S., Steiger S., Przybilla M.J., Bauer K., Baumann A., Hemmer S., Rehnitz C., Luckerath C., Sachpekidis C., Mechtersheimer G., Haberkorn U., Dimitrakopoulou-Strauss A., Reichert P., Barlogie B., Muller-Tidow C., Goldschmidt H., Hillengass J., Rasche L., Haas S.F., van Rhee F., Rippe K., Raab M.S., Sauer S., Weinhold N. (2023). Resolving the spatial architecture of myeloma and its microenvironment at the single-cell level. Nat. Commun..

[29] Dhodapkar M.V. (2024). Immune status and selection of patients for immunotherapy in myeloma: a proposal. Blood Adv..

[30] Bailur J.K., McCachren S.S., Doxie D.B., Shrestha M., Pendleton K., Nooka A.K., Neparidze N., Parker T.L., Bar N., Kaufman J.L., Hofmeister C.C., Boise L.H., Lonial S., Kemp M.L., Dhodapkar K.M., Dhodapkar M.V. (2019). Early alterations in stem-like/resident T cells, innate and myeloid cells in the bone marrow in preneoplastic gammopathy. JCI Insight.

[31] Zavidij O., Haradhvala N.J., Mouhieddine T.H., Sklavenitis-Pistofidis R., Cai S., Reidy M., Rahmat M., Flaifel A., Ferland B., Su N.K., Agius M.P., Park J., Manier S., Bustoros M., Huynh D., Capelletti M., Berrios B., Liu C.J., He M.X., Braggio E., Fonseca R., Maruvka Y.E., Guerriero J.L., Goldman M., Van Allen E.M., McCarroll S.A., Azzi J., Getz G., Ghobrial I.M. (2020). Single-cell RNA sequencing reveals compromised immune microenvironment in precursor stages of multiple myeloma. Nat. Can..

[32] de Jong M.M.E., Kellermayer Z., Papazian N., Tahri S., Hofste Op Bruinink D., Hoogenboezem R., Sanders M.A., van de Woestijne P.C., Bos P.K., Khandanpour C., Vermeulen J., Moreau P., van Duin M., Broijl A., Sonneveld P., Cupedo T. (2021). The multiple myeloma microenvironment is defined by an inflammatory stromal cell landscape. Nat. Immunol..

[33] Robling A.G., Bonewald L.F. (2020). The osteocyte: new insights. Annu. Rev. Physiol..

[34] Sabol H.M., Anloague A., Kaur J., Bustamante-Gomez C., Khan S., Paxton B.C., Nester M.R., Hackney J., Diaz-delCastillo M., Mann D., Stambough J.B., Barnes C.L., Ambrogini E., Frontier A., Ebetino F.H., Naqvi S., van Rhee F., Wardell C.P., Drake M.T., Nookaew I., Schinke C., Zangari M., Delgado-Calle J. (2025). Healing of lytic lesions and restoration of bone health in multiple myeloma through sclerostin inhibition. Exp. Hematol. Oncol..

[35] Anloague A., Delgado-Calle J. (2023). Osteocytes: new kids on the block for Cancer in bone therapy. Cancers (Basel).

[36] Delgado-Calle J., Bellido T., Roodman G.D. (2014). Role of osteocytes in multiple myeloma bone disease. Curr. Opin. Support. Palliat. Care.

[37] Delgado-Calle J., Anderson J., Cregor M.D., Hiasa M., Chirgwin J.M., Carlesso N., Yoneda T., Mohammad K.S., Plotkin L.I., Roodman G.D., Bellido T. (2016). Bidirectional notch Signaling and osteocyte-derived factors in the bone marrow microenvironment promote tumor cell proliferation and bone destruction in multiple myeloma. Cancer Res..

[38] Sabol H.M., Amorim T., Ashby C., Halladay D., Anderson J., Cregor M., Sweet M., Nookaew I., Kurihara N., Roodman G.D., Bellido T., Delgado-Calle J. (2022). Notch3 signaling between myeloma cells and osteocytes in the tumor niche promotes tumor growth and bone destruction. Neoplasia.

[39] Suvannasankha A., Tompkins D.R., Edwards D.F., Petyaykina K.V., Crean C.D., Fournier P.G., Parker J.M., Sandusky G.E., Ichikawa S., Imel E.A., Chirgwin J.M. (2015). FGF23 is elevated in multiple myeloma and increases heparanase expression by tumor cells. Oncotarget.

[40] Mulcrone P.L., Edwards S.K.E., Petrusca D.N., Haneline L.S., Delgado-Calle J., Roodman G.D. (2020). Osteocyte Vegf-a contributes to myeloma-associated angiogenesis and is regulated by Fgf23. Sci. Rep..

[41] Sabol H.M., Ashby C., Adhikari M., Anloague A., Kaur J., Khan S., Choudhury S.R., Schinke C., Palmieri M., Barnes C.L., Ambrogini E., Nookaew I., Delgado-Calle J. (2024). A NOTCH3-CXCL12-driven myeloma-tumor niche signaling axis promotes chemoresistance in multiple myeloma. Haematologica.

[42] Cheng F., Wang Z., You G., Liu Y., He J., Yang J. (2023). Osteocyte-derived exosomes confer multiple myeloma resistance to chemotherapy through acquisition of cancer stem cell-like features. Leukemia.

[43] Hemmatian H., Conrad S., Furesi G., Mletzko K., Krug J., Faila A.V., Kuhlmann J.D., Rauner M., Busse B., Jahn-Rickert K. (2021). Reorganization of the osteocyte lacuno-canalicular network characteristics in tumor sites of an immunocompetent murine model of osteotropic cancers. Bone.

[44] Naba A., Clauser K.R., Hoersch S., Liu H., Carr S.A., Hynes R.O. (2012). The matrisome: in silico definition and in vivo characterization by proteomics of normal and tumor extracellular matrices. Mol. Cell. Proteomics.

[45] Pietsch C., Engelhardt M., Ihorst G., Wystrach L., Jung J., Schmal H., Frodl A., Wäsch R., Terpos E., Herget G.W. (2025). Analysis of skeletal pain, general symptoms and patient-reported outcome measures and their value in detecting symptomatic progression – an interdisciplinary prospective study in patients with multiple myeloma. J. Bone Oncol..

[46] Cadieux B., Coleman R., Jafarinasabian P., Lipton A., Orlowski R.Z., Saad F., Scagliotti G.V., Shimizu K., Stopeck A. (2022). Experience with denosumab (XGEVA®) for prevention of skeletal-related events in the 10 years after approval. J. Bone Oncol..

[47] Raje N., Terpos E., Willenbacher W., Shimizu K., García-Sanz R., Durie B., Legieć W., Krejčí M., Laribi K., Zhu L., Cheng P., Warner D., Roodman G.D. (2018). Denosumab versus zoledronic acid in bone disease treatment of newly diagnosed multiple myeloma: an international, double-blind, double-dummy, randomised, controlled, phase 3 study. Lancet Oncol..

[48] Raje N., Vescio R., Montgomery C.W., Badros A., Munshi N., Orlowski R., Hadala J.T., Warsi G., Argonza-Aviles E., Ericson S.G., Anderson K.C. (2016). Bone marker–directed dosing of zoledronic acid for the prevention of skeletal complications in patients with multiple myeloma: results of the Z-MARK study. Clin. Cancer Res..

[49] Qiang Y.-W., Hu B., Chen Y., Zhong Y., Shi B., Barlogie B., Shaughnessy J.D. (2009). Bortezomib induces osteoblast differentiation via Wnt-independent activation of β-catenin/TCF signaling. Blood.

[50] Nierste B.A., Glackin C.A., Kirshner J. (2014). Dkk-1 and IL-7 in plasma of patients with multiple myeloma prevent differentiation of mesenchymal stem cells into osteoblasts. Am. J. Blood Res..

[51] Zangari M., Berno T., Salama M.E., Sana S., Talamo G., Pena K., Miller S., Zhan F. (2013). Effect of low dose bortezomib on bone formation in Smoldering myeloma patients. Blood.

[52] Croucher P., Dimopoulos M.A., Vervessou E.C., Verrou E., Katodritou E., Pouli A., Anagnostopoulos A., Chantry A., Zervas K., Rahemtulla A., Heath D., Terpos E. (2006). Bortezomib reduces serum Dickkopf-1 and RANKL concentrations and normalizes indices of bone remodeling in patients with relapsed multiple myeloma. Blood.

[53] Capalbo S., Chiefa A., Delia M., Diomede D., Liso V. (2009). Effective combination therapy of bortezomib and dexamethasone for a plasma cell leukemia patient with multiple osteolytic lesions and extramedullary involvement. Acta Oncol..

[54] Terpos E., Christoulas D., Kastritis E., Katodritou E., Papatheodorou A., Pouli A., Kyrtsonis M.C., Michalis E., Papanikolaou X., Gkotzamanidou M., Koulieris E., Gavriatopoulou M., Zervas K., Dimopoulos M.A., G. Greek Myeloma Study (2014). The combination of lenalidomide and dexamethasone reduces bone resorption in responding patients with relapsed/refractory multiple myeloma but has no effect on bone formation: final results on 205 patients of the Greek myeloma study group. Am. J. Hematol..

[55] Sezer O., Beksac M., Hajek R., Sucak G., Cagirgan S., Linkesch W., Meltem Akay O., Gülbas Z., Nahi H., Plesner T., Snowden J.A., Timurağaoğlu A., Dechow T., Lang A., Tuğlular T., Drach J., Armbrecht G., Potamianou A., Couturier C., Olie R.A., Feys C., Allietta N., Terpos E. (2017). Effects of single-agent bortezomib as post-transplant consolidation therapy on multiple myeloma-related bone disease: a randomized phase II study. Br. J. Haematol..

[56] Terpos E., Kastritis E., Christoulas D., Eleutherakis-Papaiakovou E., Gavriatopoulou M., Kalapanida D., Bakogeorgos M., Roussou M., Dimopoulos M.A. (2014). The combination of bortezomib and lenalidomide (VR) consolidation post-ASCT, in the absence of dexamethasone and bisphosphonates, improves response rates and bone metabolism in newly diagnosed patients with multiple myeloma. Blood.

[57] Terpos E., Christoulas D., Kastritis E., Roussou M., Migkou M., Eleutherakis-Papaiakovou E., Gavriatopoulou M., Gkotzamanidou M., Kanellias N., Manios E., Papadimitriou C., Dimopoulos M.A. (2013). VTD consolidation, without bisphosphonates, reduces bone resorption and is associated with a very low incidence of skeletal-related events in myeloma patients post ASCT. Leukemia.

[58] Hurchla M.A., Garcia-Gomez A., Hornick M.C., Ocio E.M., Li A., Blanco J.F., Collins L., Kirk C.J., Piwnica-Worms D., Vij R., Tomasson M.H., Pandiella A., San Miguel J.F., Garayoa M., Weilbaecher K.N. (2012). The epoxyketone-based proteasome inhibitors carfilzomib and orally bioavailable oprozomib have anti-resorptive and bone-anabolic activity in addition to anti-myeloma effects. Leukemia.

[59] Terpos E., Ntanasis-Stathopoulos I., Katodritou E., Kyrtsonis M.-C., Douka V., Spanoudakis E., Papatheodorou A., Eleutherakis-Papaiakovou E., Kanellias N., Gavriatopoulou M., Makras P., Kastritis E., Dimopoulos M.A. (2021). Carfilzomib improves bone metabolism in patients with advanced relapsed/refractory multiple myeloma: results of the CarMMa study. Cancers.

[60] Li Y., Li J., Zhuang W., Wang Q., Ge X., Zhang X., Chen P., Fu J., Li B. (2014). Carfilzomib promotes the osteogenic differentiation potential of mesenchymal stem cells derived from myeloma patients by inhibiting notch1 activity in vitro. Leuk. Res..

[61] Suvannasankha A., Abonour R., Farag S., Silbermann R.W., Wongsaengsak S., Cangany M.H., Rush-Taylor A., Tann M., Althouse S.K., Perkins S.M., Roodman G.D. (2017). Phase 2 study of carfilzomib and bone metabolism in patients with relapsed multiple myeloma. Blood.

[62] Gavriatopoulou M., Terpos E., Ntanasis-Stathopoulos I., Malandrakis P., Eleutherakis-Papaiakovou E., Papatheodorou A., Kanellias N., Migkou M., Fotiou D., Dialoupi I., Roussou M., Kokkali N.-A., Kastritis E., Dimopoulos M.A. (2020). Consolidation with carfilzomib, lenalidomide, and dexamethasone (KRd) following ASCT results in high rates of minimal residual disease negativity and improves bone metabolism, in the absence of bisphosphonates, among newly diagnosed patients with multiple myeloma. Blood Cancer J..

[63] Tibullo D., Longo A., Vicario N., Romano A., Barbato A., Di Rosa M., Barbagallo I., Anfuso C.D., Lupo G., Gulino R., Parenti R., Li Volti G., Palumbo G.A., Di Raimondo F., Giallongo C. (2020). Ixazomib improves bone Remodeling and counteracts sonic hedgehog Signaling inhibition mediated by myeloma cells. Cancers.

[64] Diaz-delCastillo M., Gundesen M.T., Andersen C.W., Nielsen A.L., Møller H.E.H., Vinholt P.J., Asmussen J.T., Kristensen I.B., Nyvold C.G., Abildgaard N., Levin Andersen T., Lund T. (2020). Increased bone volume by Ixazomib in multiple myeloma: 3-month results from an open label phase 2 study. J. Bone Miner. Res..

[65] Levring M.B., Diaz-delCastillo M., Gundesen M.T., Cédile O., Andersen C.W., Nielsen A.L., Møller H.E.H., Vinholt P.J., Asmussen J.T., Kristensen I.B., Nyvold C.G., Kassem M., Abildgaard N., Andersen T.L., Lund T. (2025). Ixazomib treatment has a dual effect on bone remodeling in patients with multiple myeloma: follow-up results from a phase 2 clinical study. Bone.

[66] Terpos E., Ntanasis-Stathopoulos I., Kastritis E., Hatjiharissi E., Katodritou E., Eleutherakis-Papaiakovou E., Verrou E., Gavriatopoulou M., Leonidakis A., Manousou K., Delimpasi S., Malandrakis P., Kyrtsonis M.-C., Papaioannou M., Symeonidis A., Dimopoulos M.-A. (2022). Daratumumab improves bone turnover in relapsed/refractory multiple myeloma; phase 2 study “REBUILD”. Cancers.

[67] Li X., Wang H., Li Y., Zhou J., Yang N., Wei X., Cai X., Xiao Z., Du J., Zeng H. (2025). The effects of daratumumab on bone disease in patients with newly diagnosed multiple myeloma: a multi-center retrospective study. BMC Cancer.

[68] Sonneveld P., Dimopoulos M.A., Boccadoro M., Quach H., Ho P.J., Beksac M., Hulin C., Antonioli E., Leleu X., Mangiacavalli S., Perrot A., Cavo M., Belotti A., Broijl A., Gay F., Mina R., Nijhof I.S., van de Donk N.W.C.J., Katodritou E., Schjesvold F., Sureda Balari A., Rosiñol L., Delforge M., Roeloffzen W., Silzle T., Vangsted A., Einsele H., Spencer A., Hajek R., Jurczyszyn A., Lonergan S., Ahmadi T., Liu Y., Wang J., Vieyra D., van Brummelen E.M.J., Vanquickelberghe V., Sitthi-Amorn A., de Boer C.J., Carson R., Rodriguez-Otero P., Bladé J., Moreau P. (2024). Daratumumab, Bortezomib, lenalidomide, and dexamethasone for multiple myeloma. N. Engl. J. Med..

[69] Corre J., Vincent L., Moreau P., Hebraud B., Hulin C., Béné M.C., Broijl A., Caillot D., Delforge M., Dejoie T., Facon T., Lambert J., Leleu X., Macro M., Perrot A., Zweegman S., Filleron T., Cabarrou B., van de Donk N.W.C.J., Mahéo S., Hua W., Wang J., Krevvata M., Vanquickelberghe V., de Boer C., Tuozzo A., Borgsten F., Rowe M., Carson R., Wuilleme S., Sonneveld P. (2025). Daratumumab-bortezomib-thalidomide-dexamethasone for newly diagnosed myeloma: CASSIOPEIA minimal residual disease results. Blood.

[70] Moreau P., Hulin C., Perrot A., Arnulf B., Belhadj K., Benboubker L., Zweegman S., Caillon H., Caillot D., Avet-Loiseau H., Delforge M., Dejoie T., Facon T., Sonntag C., Fontan J., Mohty M., Jie K.-S., Karlin L., Kuhnowski F., Lambert J., Leleu X., Macro M., Orsini-Piocelle F., Roussel M., Schiano de Colella J.M., van de Donk N.W.C.J., Wuillème S., Broijl A., Touzeau C., Tiab M., Marolleau J.-P., Meuleman N., Vekemans M.-C., Westerman M., Klein S.K., Levin M.-D., Offner F., Escoffre-Barbe M., Eveillard J.-R., Garidi R., Hua W., Wang J., Tuozzo A., de Boer C., Rowe M., Vanquickelberghe V., Carson R., Vermeulen J., Corre J., Sonneveld P. (2024). Bortezomib, thalidomide, and dexamethasone with or without daratumumab and followed by daratumumab maintenance or observation in transplant-eligible newly diagnosed multiple myeloma: long-term follow-up of the CASSIOPEIA randomised controlled phase 3 trial. Lancet Oncol..

[71] Ritchie D., Colonna M. (2017). Mechanisms of action and clinical development of Elotuzumab. Clin. Transl. Sci..

[72] Inoue Y., Tenshin H., Teramachi J., Sumitani R., Oda A., Maeda Y., Oura M., Sogabe K., Maruhashi T., Takahashi M., Fujii S., Nakamura S., Miki H., Hara T., Endo I., Kagawa K., Ozaki S., Hiasa M., Harada T., Abe M. (2024). Elotuzumab-mediated ADCC with Th1-like Vγ9Vδ2 T cells to disrupt myeloma–osteoclast interaction. Cancer Sci..

[73] Merz M., Jurgens E., Fandrei D., Usmani S.Z., Mailankody S. (2026). Pharmacokinetics of CAR T cells in multiple myeloma – the good, the bad, and the ugly. Leukemia.

[74] Sun F., Cheng Y., Chen J.-R., Wanchai V., Mery D.E., Xu H., Gai D., Al Hadidi S., Schinke C., Thanendrarajan S., Zangari M., van Rhee F., Tricot G., Shaughnessy J.D., Zhan F. (2023). BCMA- and CST6-specific CAR T cells lyse multiple myeloma cells and suppress murine osteolytic lesions. J. Clin. Invest..

[75] Ussmann J., Fischer L., Born P., Grieb N., Hoffmann S., Baber R., Wang S.-Y., Kloetzer C., Heyn S., Herling M., Herling C., Jentzsch M., Franke G.-N., Hofbauer L., Platzbecker U., Vucinic V., Weidner H., Merz M. (2023). Increased bone turnover and decreased BCMA levels in patients with response to anti-BCMA CAR T cell therapy in relapsed/refractory multiple myeloma. Blood.

[76] Silbermann R., Roodman G.D. (2013). Myeloma bone disease: pathophysiology and management. J. Bone Oncol..

[77] Hofbauer L.C., Bozec A., Rauner M., Jakob F., Perner S., Pantel K. (2021). Novel approaches to target the microenvironment of bone metastasis. Nat. Rev. Clin. Oncol..

[78] Terpos E., Ntanasis-Stathopoulos I., Gavriatopoulou M., Dimopoulos M.A. (2018). Pathogenesis of bone disease in multiple myeloma: from bench to bedside. Blood Cancer J..

[79] Born P., Fandrei D., Wang S.Y., Perez-Fernandez C., Fischer L., Ussmann J., Bach E., Hoffmann S., Metzeler K.H., Herling M., Herling C., Jentzsch M., Boldt A., Seiffert S., Baber R., Weidner H., Franke G.-N., Denecke T., Sabri O., Platzbecker U., Vucinic V., Meyer H.J., Kurch L., Merz M. (2025). Prognostic significance of PET/CT for CAR T cell therapy in relapsed/refractory multiple myeloma. HemaSphere.

[80] Fischer L., Grieb N., Born P., Weiss R., Seiffert S., Boldt A., Fricke S., Franz P., Heyn S., Kubasch A.S., Baber R., Weidner H., Wang S.Y., Bach E., Hoffmann S., Ussmann J., Kirchberg J., Hell S., Schwind S., Metzeler K.H., Herling M., Jentzsch M., Franke G.-N., Sack U., Reiche K., Köhl U., Platzbecker U., Vucinic V., Merz M. (2024). Cellular dynamics following CAR T cell therapy are associated with response and toxicity in relapsed/refractory myeloma. Leukemia.

[81] Rade M., Fandrei D., Kreuz M., Seiffert S., Grahnert A., Friedrich M., Wiemers T., Born P., Fischer L., Weidner H., Hofbauer L.C., Baber R., Wang S.Y., Bach E., Hoffmann S., Scolnick J., Friedrich M., Keramati F., Brazda P., Sebestyen Z., Kuball J., Alb M., Scheller L., Hudecek M., Einsele H., Metzeler K.H., Herling M., Herling C.D., Jentzsch M., Franke G.-N., Boldt A., Köhl U., Platzbecker U., Vucinic V., Reiche K., Merz M. (2026). A longitudinal single-cell atlas to predict outcome and toxicity after BCMA-directed CAR T cell therapy in multiple myeloma. Cancer Cell.

[82] Rade M., Grieb N., Weiss R., Sia J., Fischer L., Born P., Boldt A., Fricke S., Franz P., Scolnick J., Venkatraman L., Xu S., Kloetzer C., Heyn S., Kubasch A.S., Baber R., Wang S.Y., Bach E., Hoffmann S., Ussmann J., Schetschorke B., Hell S., Schwind S., Metzeler K.H., Herling M., Jentzsch M., Franke G.-N., Sack U., Köhl U., Platzbecker U., Reiche K., Vucinic V., Merz M. (2024). Single-cell multiomic dissection of response and resistance to chimeric antigen receptor T cells against BCMA in relapsed multiple myeloma. Nat. Can..

[83] Tang L., Pan S., Wei X., Xu X., Wei Q. (2023). Arming CAR-T cells with cytokines and more: innovations in the fourth-generation CAR-T development. Mol. Ther..

